# Cytotoxicity Evaluation of Photosensitizer-Conjugated Hexagonal Upconverting Nanoparticles

**DOI:** 10.3390/nano13091535

**Published:** 2023-05-03

**Authors:** Mykhailo Nahorniak, Viktoriia Oleksa, Taras Vasylyshyn, Ognen Pop-Georgievski, Eliška Rydvalová, Marcela Filipová, Daniel Horák

**Affiliations:** Institute of Macromolecular Chemistry, Czech Academy of Sciences, 160 00 Prague, Czech Republic; nahorniak@imc.cas.cz (M.N.); oleksa@imc.cas.cz (V.O.); vasylyshyn@imc.cas.cz (T.V.); georgievski@imc.cas.cz (O.P.-G.); rydvalova@imc.cas.cz (E.R.); filipova@imc.cas.cz (M.F.)

**Keywords:** upcoverting, nanoparticles, rose bengal, photosensitizer, cytotoxicity

## Abstract

In this report, we synthesized hexagonal NaYF_4_:Yb,Er upconverting nanoparticles (UCNPs) of 171 nm in size with a narrow particle size distribution. To address their colloidal stabi-lity in aqueous media and to incorporate a photosensitizer that can produce reactive singlet oxygen (^1^O_2_) to kill tumor cells, UCNPs were conjugated with 6-bromohexanoic acid-functionalized Rose Bengal (RB) and coated with PEG-alendronate (PEG-Ale). The particles were thoroughly characterized by transmission electron microscopy, dynamic light scattering, ATR FTIR, X-ray photoelectron spectroscopy, thermogravimetric analysis, and spectrofluorometry, and ^1^O_2_ formation was detected using a 9,10-diphenylanthracene spectrophotometric probe. Cytotoxicity determination on rat mesenchymal stem cells by using the MTT assay showed that neutralization of the large positive surface charge of neat UCNPs with PEG-Ale and the bound RB sensitizer significantly reduced the concentration-dependent cytotoxicity. The presented strategy shows great potential for the use of these particles as a novel agent for the photodynamic therapy of tumors.

## 1. Introduction

Upconverting nanoparticles (UCNPs) with sizes comparable to those of proteins, genes, or viruses offer many interesting properties for biomedical applications, especially in multimodal biological imaging, photodynamic therapy (PDT), drug delivery, and antibacterial treatment [1,2,3]. Anti-inflammatory or photocatalytic performance and the subsequent bacteria-killing performance using electron transfer mechanisms were described in [4,5]. These applications place stringent requirements on the physicochemical and pharmacological properties of the particles, including their chemical composition, crystal structure, monodisperse size, upconversion behavior, charge, chemical and colloidal stability without particle aggregation, surface structure, and biocompatibility (non-toxicity). The ability to control the particle size (typically in the range of 20–200 nm) and thus the surface-to-volume ratio is important here. In particular, small nanoparticles improve diffusion in tissues and allow effective drug targeting. Upconversion properties of UCNPs enable them to absorb typically two or more photons of relatively low energy in the infrared region and convert them into a single emitted photon of higher energy in the visible or ultraviolet region [6]. The attractiveness of UCNPs, which consist of rare earth lanthanide-based transition metals, typically Ln^3 +^, Yb^3 +^, Tm^3 +^, and Er^3 +^, lies in their good internalization by cells, little damage to healthy cells, and NIR penetration deep into tissue; the particles also provide long luminescence lifetimes and little background noise and autofluorescence [7,8]. In addition, they exhibit high photostability and diverse excitation wavelengths, as well as tunable emission wavelengths, no photoblinking or photobleaching, and have a large surface area that can be used for conjugation with various biomolecules. The disadvantage is the relatively low quantum yield of photoluminescence.

UCNPs are synthesized by various high-temperature methods such as coprecipitation, thermal decomposition, and the hydrothermal/solvothermal method and in most cases are coated with oleic acid. Depending on the reaction conditions, the size and morphology of the nanoparticles, which can be spherical, cubic, and hexagonal, can be controlled, which is closely related to the crystalline phase of the particles. Particles with a size <20 nm are generally spherical, while particles with a size >20 nm exhibit the shape of cubes or hexagonal plates [9]. The hexagonal lattice structure of UCNPs enables efficient energy transfer between rare earth ions, leading to high upconversion efficiency. At the same time, UCNPs applicable in biomedicine should be colloidally stable and form aqueous dispersions. Therefore, surface engineering with polymers is necessary to transfer particles to water, prevent degradation, and ensure biocompatibility; at the same time, particles should not lose their high upconversion and luminescence intensity. Functionalization techniques include ligand exchange, oxidation, layer-by-layer assembly, “grafting-on” and “grafting-from” methods, encapsulation, and silanization or modification with various reactive polymers based on poly(ethylene glycol) (PEG), polyethylenimine, poly(*N*-vinylpyrrolidone), poly(acrylic acid), poly(amidoamine), poly(*N*-(2-hydroxypropyl)methacrylamide), dextran, poly(methyl vinyl ether-*co*-maleic acid), silica, etc. [10,11,12]. Reactive functional groups include amino, carboxyl, phosphate, and sulfhydryl groups, which are useful for binding biological molecules and/or photosensitizers (PSs), which are essential for the PDT of tumors and the killing of bacteria. An example of a PS is Rose Bengal (RB), whose excitation allows the high production of reactive oxygen species such as singlet oxygen, which kills cells and bacteria [13]. Selective modification of the UCNP surface with RB while preserving the colloidal stability of the particles and at the same time protecting them from non-specific interactions with the biological environment is foreseen as an important step toward the implementation of PDT.

In this paper, we have prepared an RB derivative, namely a 6-bromohexanoic-acid-modified RB photosensitizer, which was conjugated to Ale-PEG-stabilized hexagonal UCNPs. The cytotoxicity of the RB-conjugated nanoparticles was determined and singlet oxygen formation was detected.

## 2. Experimental

### 2.1. Chemicals

Erbium(III) chloride hexahydrate (98%), anhydrous yttrium(III) and ytterbium(III) chlorides, 2-ethoxy-1-ethoxycarbonyl-1,2-dihydroquinoline (EEDQ; 99%), 6-bromohexanoic acid (97%), Rose Bengal disodium salt (RB; 95%), sodium alendronate (Ale; 99%), sodium hydroxide (99%), ammonium fluoride (99.9%), octadec-1-ene (OD; 90%), and 9,10-diphenylanthracene (DPA; analytical standard) were purchased from Sigma-Aldrich (St. Louis, MO, USA). Gibco™ Dulbecco’s modified Eagle’s medium (DMEM) was obtained from Thermo Fisher Scientific (Waltham, MA, USA). The [3-(4,5-dimethylthiazol-2-yl)-2,5-diphenyltetrazolium bromide] (MTT) assay was purchased from Abcam (Cambridge, UK). Oleic acid (OA; 98%) and the other solvents were from Lachema (Brno, Czech Republic). PEG_5,000_-alendronate (PEG-Ale) was prepared as described in the previous article [14]. Absolute ethanol and other chemicals were purchased from LachNer (Neratovice, Czech Republic), while the cellulose dialysis membranes (MWCO 14 and 100 kDa) were from Spectrum Europe (Breda, The Netherlands). Water was purified on a Milli-Q IQ 7000 column from Millipore (Molsheim, France).

### 2.2. Cell Line

Rat mesenchymal stem cells (rMSCs; kindly provided by the Institute of Experimental Medicine, Czech Academy of Sciences, Prague) were cultivated in DMEM containing 10% fetal bovine serum and 1% penicillin–streptomycin. The cells were maintained at 37 °C in a 5% CO_2_ humidified atmosphere. The cells were subcultured with trypsin-EDTA solution (Thermo Fisher Scientific).

### 2.3. Synthesis of NaYF_4_:Yb,Er Nanoparticles (UCNPs)

For the preparation of UCNPs, lanthanide chlorides (YCl_3_/YbCl_3_/ErCl_3_·6H_2_O = 1.56:0.4:0.04 mol/mol/mol) were mixed with OA (6 mL) and OD (15 mL) under heating (160 °C) and magnetic stirring for 30 min in an inert atmosphere (Ar). The mixture was cooled to room temperature (RT) and a methanolic dispersion of NaOH and NH_4_F (2.5/4 mol/mol; 10 mL) was added and the mixture was heated to 160 °C under vacuum (7.5 kPa) and refluxed at 300 °C for 90 min while stirring. The resulting UCNP@OA particles were isolated by centrifugation (3460 rcf) for 30 min and washed four times with a hexane/ethanol (1/4 *v*/*v*) mixture. Before particle surface modification, the UCNP@OA particles were thoroughly washed three times with water/ethanol (the water gradually displaced the ethanol) and separated by centrifugation to obtain neat UCNPs.

### 2.4. Synthesis of 2-[(5-Carboxypentyloxy)carbonyl]-3,4,5,6-tetrachlorophenyl-Rose Bengal (RB-CPC)

**Figure 1:**
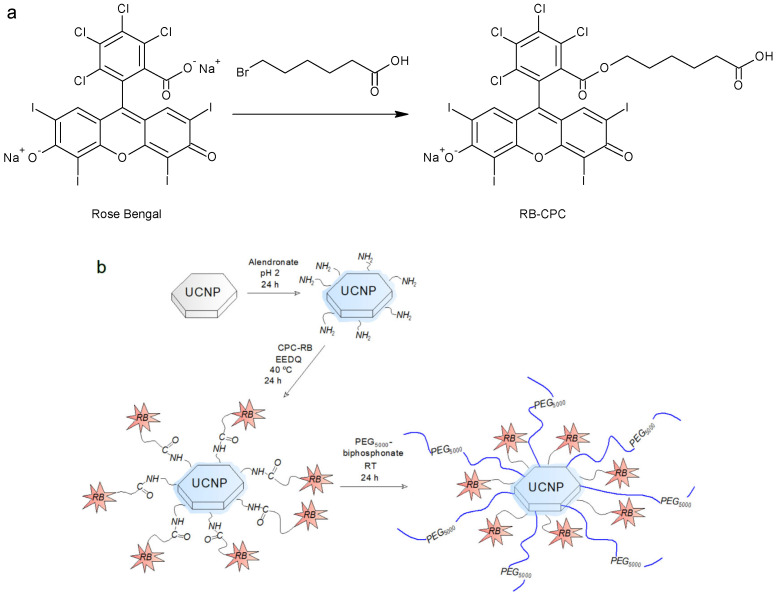
(**a**) Preparation of 2-[(5-carboxypentyloxy)carbonyl]- rose bengal (RB-CPC) and (**b**) modification of hexagonal UCNP with RB-CPC and PEG-Ale.

RB-CPC was obtained through the reaction of RB with 6-bromohexanoic acid (Figure 1a). In a 10 mL round-bottom flask, RB (0.2 mmol) and 6-bromohexanoic acid (0.62 mmol) were dissolved in dimethylformamide (DMF; 2.5 mL) and the solution was heated at 80 °C for 7 h under reflux with magnetic stirring (600 rpm). DMF was removed on a rotary evaporator at 45 °C under reduced pressure (1 kPa). The dry RB-CPC was washed three times with diethyl ether and water and lyophilized to give a reaction yield of 82.5%; the product quality was analyzed by ^1^H NMR spectroscopy (Figure S1).

### 2.5. Synthesis of UCNP@Ale-RB-CPC/Ale-PEG

The reaction of UCNPs with Ale, RB-CPC, and PEG-Ale is shown on Figure 1b. Ale (100 mg) was acidified by titration with 0.1 M HCl to pH 2 as previously reported [15] and added to an aqueous dispersion of UCNPs (130 mg; 5 mL) with stirring at RT for 48 h. The resulting alendronate-functionalized upconverting particles (UCNP@Ale) were separated by centrifugation (3460 rcf) for 30 min, the sediment was dispersed in water, purified by dialysis against water with a cellulose membrane (MWCO 14 kDa) for 48 h, and lyophilized. The washed UCNP@Ale particles (35 mg) were then mixed with RB-CPC (0.01 mmol) and EEDQ (0.013 mmol) in DMF (2 mL), the reaction was run at 40 °C for 24 h, and the product was dialyzed (MWCO 100 kDa) against water/methanol (1/1 *v*/*v*) and water for 24 h each time. Finally, the particles (12 mg) were mixed with aqueous PEG-Ale solution (3 mL) at RT for 24 h under stirring (400 rpm) and the mixture was dialyzed (MWCO 100 kDa) against water for 24 h to form UCNP@Ale-RB-CPC/Ale-PEG particles.

### 2.6. Detection of Singlet Oxygen Generation

A mixture of ethanolic solutions of DPA (2 × 10^−5^ mol/L) and RB or RB-CPC or UCNP@Ale-RB-CPC/Ale-PEG (1.62 × 10^−9^ mol/L) was irradiated in the dark with an LED lamp (525–535 nm; 0.16·10^−3^ W/mm^2^) and a 980 nm laser (MDL-III-980-2W; 0.7 W/mm^2^), respectively [16]. DPA absorbance was measured at 330–410 nm every 10 min by UV-Vis spectrophotometry and the decrease in DPA absorbance reflected singlet oxygen production.

### 2.7. Characterization of Nanoparticles

The morphology of the particles was studied with a Tecnai G2 Spirit Twin 12 transmission electron microscope (TEM; FEI; Brno, Czech Republic) [14]. The particle size and distribution were determined from at least 500 objects on 6 different TEM images using ImageJ 1.52a software (National Institutes of Health; Bethesda, MD, USA). The number-average diameter (*D*_n_), weight-average diameter (*D*_w_), and dispersity (*Ð*) were obtained according to the following equations:

*D*_n_ = ∑N_i_*D*_i_/∑N_i_ (1)

*D*_w_ = ∑N_i_*D*_i_^4^/∑N_i_*D*_i_^3^ (2)

*Ð* = *D*_w_/*D*_n_ (3)

where N_i_ and *D*_i_ are the number and diameter of the particle, respectively.

Dynamic light scattering (DLS) was measured in water at 25 °C on a ZEN 3600 Zetasizer Nano Instrument (Malvern Instruments; Malvern, UK). The hydrodynamic diameter (*D*_h_) and polydispersity (*PD*) were calculated from the intensity-weighted distribution obtained by CONTIN correlation function analysis in Malvern software. ^1^H NMR spectra were measured at 25 °C on a Bruker Avance III 600 spectrometer (Rheinstetten, Germany) equipped with a 5 mm diffusion probe-head with a 90° pulse (18 μs width), a relaxation delay of 10 s, a spectral width of 7812 Hz, an acquisition time of 4.19 s, and 64 scans. ATR FTIR spectra were measured on a Nexus Nicolet 870 FTIR spectrometer (Madison, WI, USA) equipped with a liquid-nitrogen-cooled mercury cadmium telluride detector using a GoldenGate single reflection diamond ATR system (Specac; Orpington, UK). Thermogravimetric analysis (TGA) was determined in the air between 25 and 700 °C at a heating rate of 10 °C/min on a PerkinElmer TGA 7 analyzer (Norwalk, CT, USA). Luminescence spectra were measured on an FS5 spectrofluorometer (Edinburgh Instruments; Edinburgh, UK) coupled with a 5 × 8 mm^2^ MDL-III-980-2W CW 980 nm laser diode (excitation source). The excitation curves of all UCNPs were normalized by the red peak area (630–690 nm). A K-Alpha^+^ XPS spectrometer (Thermo Fisher Scientific; East Greenstead, UK) operating at reduced pressure (1 × 10^−7^ Pa) and Thermo Advantage software were used to determine the X-ray photoelectron spectra (XPS). Particles spread on a conductive carbon tape were analyzed using microfocused (spot size 400 μm) and monochromated Al Kα X-rays (200 eV for survey and 50 eV for high-energy resolution). The following parameters were used: an X-ray incidence angle of 30°, a normal emission angle along the surface and a dual-charge compensation system for electrons, and low energy Ar^+^ ions. The analyzer transmission function, Scofield sensitivity factors, and effective photoelectron attenuation lengths were calculated using the standard TPP-2 M formalism. The binding energy scale of the spectrometer was calibrated by the known positions of the C 1s C–C and C–H and C–O and C(=O)–O peaks of poly(ethylene terephthalate) and the Cu 2p, Ag 3d, and Au 4f peaks of Cu, Ag, and Au, respectively. All spectra were charge-referenced to the C 1s contribution with a binding energy of 285 eV, which was assigned to the C–C and C–H bonds.

### 2.8. Determination of Cytotoxicity

The cytotoxicity of UCNPs, i.e., the metabolic activity of cells after treatment with UCNPs, was measured using the MTT assay. Upon reaction, the yellow MTT dye is converted to purple formazan in living cells by the action of mitochondrial reductases. The rMSCs (8 × 10^3^ cells/well) were seeded in 96-well flat-bottom plates (TPP; Trasadingen, Switzerland) and grown in complete culture medium (100 μL/well) for 24 h. UCNPs, UCNP@Ale-RB-CPC/PEG-Ale particles and RB were diluted to concentrations of 3.9–500 µg/mL and incubated with cells at 37 °C for 24 h. The medium was replaced with complete growth medium (100 μL) containing MTT (final concentration 500 µg/mL) and incubation was continued at 37 °C for 4 h. The MTT solution was removed, and the resulting formazan crystals were dissolved with dimethyl sulfoxide (100 µL) under shaking for 10 min. Absorbance was measured on a Synergy H1 hybrid multi-mode plate reader (Bio-Tek; Prague, Czech Republic) at 570 nm. Relative cell viability was calculated as the percentage of viable cells in each treatment compared to the control (100%). Cytotoxicity was expressed as the mean ± standard error of the mean (S.E.M.) of at least three independent experiments performed in triplicate. Statistical differences were evaluated by a two-tailed unpaired Student’s *t*-test using GraphPad Prism version 5.03 software (La Jolla, CA, USA). Values * *p* <0.05, ** *p* <0.01, and *** *p* <0.001 were considered statistically significant.

## 3. Results and Discussion

### 3.1. Synthesis and Properties of Hexagonal UCNPs

**Figure 2:**
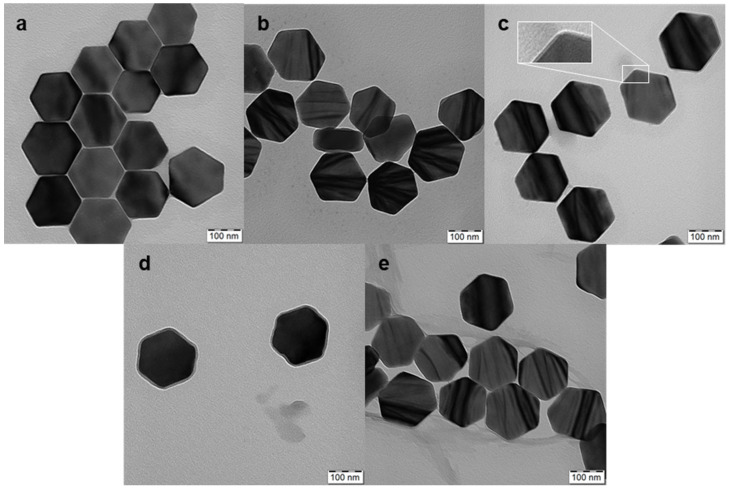
TEM micrographs of hexagonal (**a**) UCNP@OA, (**b**) UCNPs, (**c**) UCNP@Ale, (**d**) UCNP@Ale-RB-CPC, and (**e**) UCNP@Ale-RB-CPC/Ale-PEG nanoparticles. The inset shows a thin layer of Ale on the particles.

**Figure 3:**
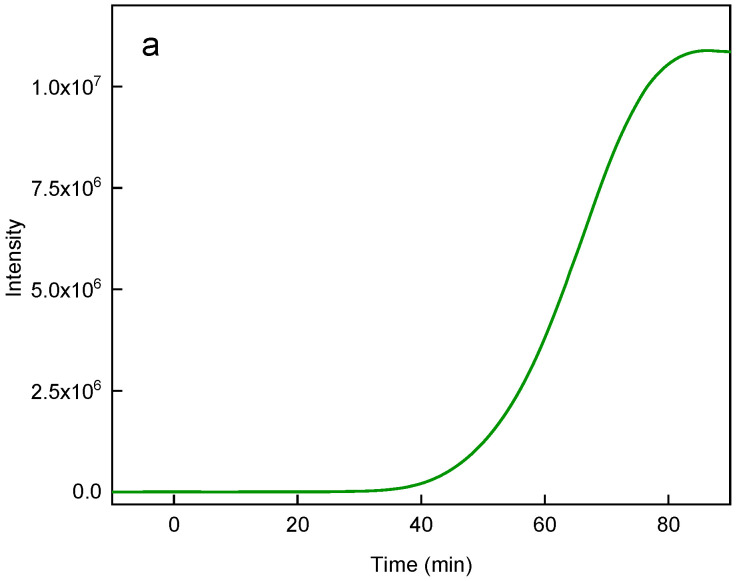

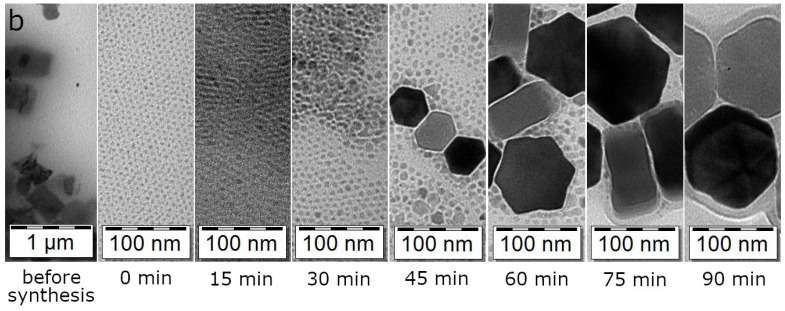
(**a**) Luminescence intensity of UCNP@OA particles excited at 980 nm and (**b**) their TEM micrographs at different reaction times.

High-temperature coprecipitation of lanthanide chlorides in octadec-1-ene solvent stabilized with oleic acid was used to prepare hexagonal UCNP@OA particles [14]. During the synthesis, it was necessary to remove methanol and water released from ErCl_3_ hexahydrate under vacuum to form hexagonal UCNPs. Their TEM micrograph revealed a hexagonal shape with an average size of *D*_n_ = 171 nm (length between the hexagon vertices), a thickness of 69 nm, and a dispersity of *Ð* = 1.01, documenting a narrow particle size distribution (Figure 2a). TEM and upconversion luminescence spectroscopy at 980 nm excitation were used to investigate the formation of hexagonal UCNPs at different times (Figure 3a,b). No particles were observed at short reaction times (<30 min). Rapidly growing nuclei due to Ostwald ripening [17] appeared after 45 min of reaction, which was accompanied by an increase in the intensity of the emitted light. Particle formation was completed only after a reaction time of 75 min was reached; from this time on, the particle morphology was stable. Therefore, we chose a 90 min reaction time as the optimal one for the synthesis of hexagonal UCNPs. It is important to note that before surface modification, the hexagonal UCNP@OA particles were thoroughly washed with ethanol/water and water to remove OA; the purified particles (with unchanged morphology) were designated as neat UCNPs (Figure 2b). Their DLS measurements in water confirmed a narrow particle size distribution, and polydispersity (*PD* = 0.04), hydrodynamic size (*D*_h_ = 228 nm), and ξ-potential (38 mV) were evaluated (Table 1). It is known that the hydrodynamic size is always larger than the size from TEM due to the hydration solvent layer on the particles, while TEM measures the dry core size. Moreover, TEM is a number-based observation, whereas DLS, based on light scattering intensity, which is proportional to the sixth power of the diameter, weights larger sizes. The highly positive ξ-potential then comes from dissociated metal cations on the particle surface.

**Figure 4:**
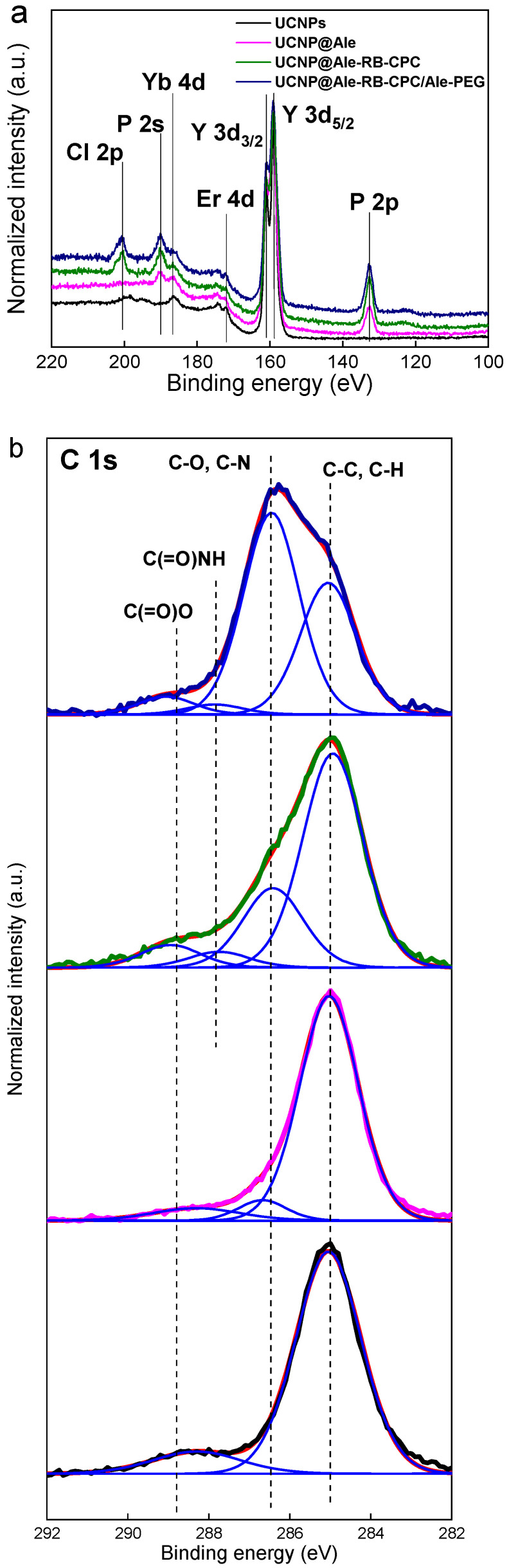
Comparison of the high-resolution XPS spectra of the initial hexagonal UCNP@OA (black), UCNP@Ale (magenta), UCNP@Ale-RB-CPC (green), and UCNP@Ale-RB-CPC/Ale-PEG (navy) in the region of (**a**) P 2p, Y 3d, Er 4d, Yb 4d, P 2s, Cl 2p, and (**b**) C 1s (red, fitted data; blue, individual contributions of functional groups on the UNCP surface).

The chemical structure of hexagonal UCNP@OA particles was confirmed by high-resolution XPS spectra. The analysis showed Y 3d (159.5 eV), Er 4d (172.5 eV), Yb 4d (186.9 eV), F 1s (686.8 eV), and Na 1s (1071.1 eV) peaks (Figure 4a; Table 2). The results are consistent with previously published data [18]. In the C 1s spectrum, two peaks originating from the C-C and C(=O)-O^–^ groups documented the presence of OA on the particle surface.

### 3.2. Surface Engineering of Hexagonal UCNPs

**Figure 5:**
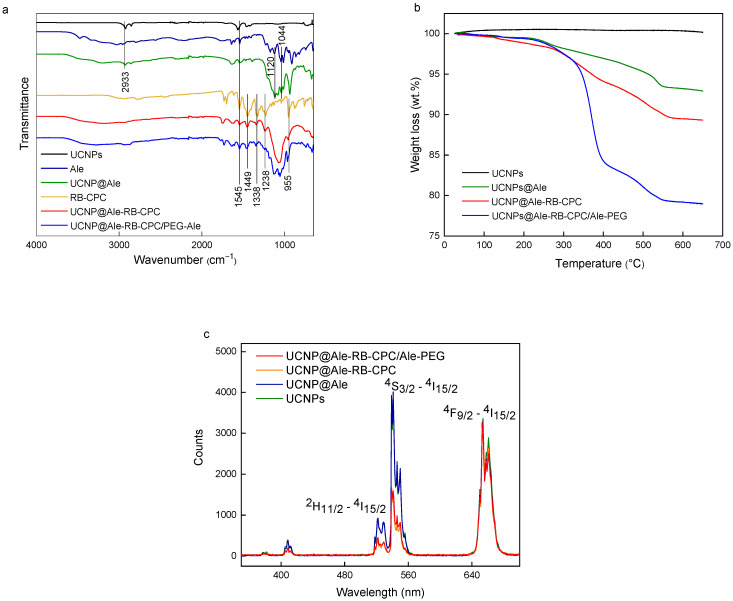
(**a**) FTIR spectra, (**b**) thermogravimetric analysis, and (**c**) luminescence spectra of surface-modified hexagonal UCNPs excited at 980 nm.

The coating of hexagonal UCNPs by RB (excitation at 525 nm) and PEG-Ale was achieved in three stages. (i) In the first step, the hexagonal UCNPs were modified with Ale containing an amino group. The TEM micrograph of the UCNP@Ale particles (Figure 2c) showed their hexagonal shape similar to that of the original UCNPs. However, a thin corona (~4 nm thick) was also visible in the micrograph, confirming the presence of Ale coating on the particle surface (see inset in Figure 2c). The DLS revealed an enhanced hydrodynamic size of 210 nm and a positive ξ-potential of 25 mV, which was lower than that of neat UCNPs due to the interaction of surface metal ions with Ale (Table 1). FTIR spectra of hexagonal UCNP@Ale showed strong bands in the region of 1200–900 cm^−1^ corresponding to C-O and P-O stretching vibrations and a peak of Ale at 900 cm^−1^ (Figure 5a). In addition, XPS spectroscopy indicated the presence of a P 2p peak at 132.4 eV originating from bisphosphonate groups. Significant changes in the C 1s spectrum were attributed to the C-O and C-N functional groups of Ale at 286.7 eV (Figure 4b; Table 2). Remarkably, the NH_2_ and NH_3_^+^ groups, as well as the corresponding peaks observed at 399.5 and 400.5 eV, respectively, were superimposed by broad contributions of Y 3s at 396 eV. Thus, all characterization methods indicated successful binding of Ale to hexagonal UCNPs.

(ii) In the second step, using EEDQ as a coupling agent, the amino groups of hexa-gonal UCNP@Ale reacted with the carboxyl groups of RB-CPC, which was obtained by reacting RB with 6-bromohexanoic acid (Figure 1). The number-average diameter remained unchanged (Figure 2d) from the previous results, while the hydrodynamic size increased to 1480 nm due to partial aggregation and the ξ-potential decreased to 10 mV due to RB-CPC binding (Table 1). The explanation for the increased hydrodynamic particle size of UCNP@Ale-RB-CPC in water lies in the low value of the ξ-potential (10 mV), which was not sufficient to electrostatically stabilize the particles; in contrast, the ξ-potential of the neat and UCNP@Ale particles was 38 and 25 mV, respectively. In addition, RB-CPC is highly hydrophobic, consequently favoring hydrophobic interactions between individual UCNP@Ale-RB-CPC particles. The effect is even stronger under DLS conditions of measurements in water. Therefore, the particles were coated with PEG in the last synthesis step to ensure their steric stabilization. ATR FTIR spectra of the hexagonal UCNP@Ale-RB-CPC particles showed the presence of the characteristic C–Cl stretching vibration at 759 cm^−1^. The peaks at 1630 and 1340 cm^−1^ arise from the asymmetric and symmetric stretching vibrations of COO, respectively (Figure 5a). The peaks at ~1550 and 1450 cm^−1^ were ascribed to the characteristic absorption of the aromatic ring vibration, while the peak at 1235 cm^−1^ corresponded to the C–O–C groups. In addition, the reaction of RB-CPC with the amino groups of hexagonal UCNP@Ale was confirmed by C 1s XPS spectra due to the appearance of the amide bond peak at 287.8 eV and the enhancement of the peak originating from the C-O, C-I, and C-Cl groups at 286.6 eV (Figure 4b); moreover, the Cl and I elements appeared in the spectra (Table 2).

(iii) In the third step, the hexagonal UCNP@Ale-RB-CPC particles were coated with PEG-Ale to achieve good colloidal stability in aqueous solutions. Indeed, it is known that the bisphosphonate groups of Ale are tightly complexed with the lanthanide ions on the particle surface [19]. TEM analysis revealed a distinct organic corona and no changes in size and morphology (Figure 2e) compared to previous particles (Figure 2d). According to DLS, the hydrodynamic size and ξ-potential of UCNP@Ale-RB-CPC/Ale-PEG particles decreased to 720 nm and 4 mV, respectively, compared with UCNP@Ale-RB-CPC, reflecting the better particle stabilization and surface charge shielded by electroneutral PEG. The presence of PEG on the particle surface was documented by ATR FTIR spectroscopy, where characteristic O-H, νC−H symmetric and asymmetric, and C-O stretching vibrations were observed at 3390, 2880, and 1106–1100 cm^−1^, respectively (Figure 5a). The mo-dification of the hexagonal UCNP@Ale-RB-CPC with PEG-Ale was also confirmed by XPS spectroscopy mainly by the appearance of a C-O peak at 286.6 eV, which is characteristic of PEG (Figure 4b; Table 2). Moreover, there was an increase in the P 2p peak at 132.4 eV, which was accompanied by a decrease in contributions originating from UCNPs. The binding of PEG-Ale to the particle surface was also demonstrated by an increase in the contributions of P and N elements (Figure 4b; Table 2) and C-O groups at 286.5 eV originating from PEG [18,20].

While TGA of hexagonal UCNP@Ale confirmed the presence of Ale at 6.7 wt.% (Fi-gure 5b), the RB-CPC and PEG-Ale contents of hexagonal UCNP@Ale-RB-CPC and UCNP@Ale-RB-CPC/Ale-PEG particles were 5.5 and 10.3 wt.%, respectively (Figure 5b). Thus, the amount of RB was almost twice lower than on small spherical UCNPs, possibly originating from the larger surface-to-volume ratio of small particles [21]. The upconversion luminescence and light absorption of hexagonal UCNPs and hexagonal UCNP@Ale-RB-CPC/Ale-PEG particles (Figure 5c) were similar to those of smaller spherical UCNPs [21]. While the luminescence intensity of neat hexagonal UCNPs and hexagonal UCNP@Ale (1 mg/mL) at 515–560 nm excitation was the same, that of hexagonal UCNP@Ale-RB-CPC and UCNP@Ale-RB-CPC/Ale-PEG was significantly decreased. A comparison of the hexagonal UCNP and UCNP@Ale-RB-CPC/Ale-PEG curves showed that 51% of the light emitted by the nanoparticles was absorbed by RB-CPC.

**Figure 6:**
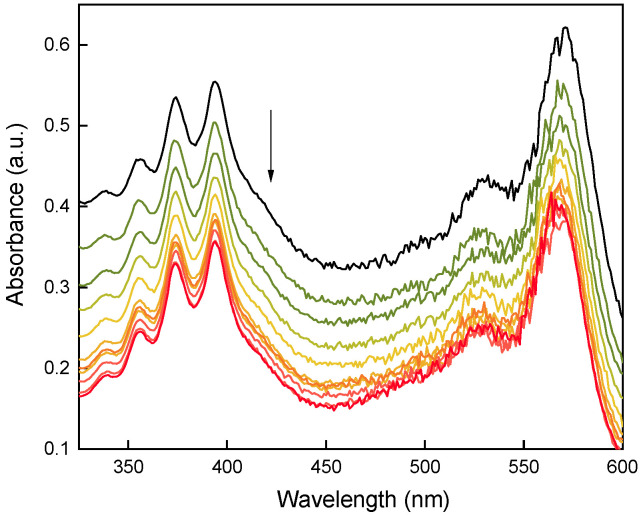
Time-dependent decrease in DPA absorbance in the presence of hexagonal UCNP@Ale-RB-CPC/Ale-PEG particles excited at 980 nm and measured by UV-Vis spectroscopy. The arrow points to the generation of ^1^O_2_. The black curve represents the beginning of the experiment; each subsequent curve was measured after 10 min.

As the proof-of-concept, the generation of ^1^O_2_ from hexagonal UCNP@Ale-RB-CPC/Ale-PEG particles was investigated using a DPA-based assay. The concentration of UCNP@Ale-RB-CPC/Ale-PEG in the solution was selected to be 1.62 × 10^−9^ mol/L considering the reasonably high upconversion luminescence and low cytotoxicity. Understandably, we could increase the UNCP concentration, but at the expense of increased cytotoxicity, which is undesirable in biological applications. The continuous decrease in DPA absorbance (at 330–410 nm) with increasing irradiation time of the 980-nm laser, which was more pronounced at the beginning of the measurements, confirmed the production of ^1^O_2_ by RB-CPC (Figure 6). The UCNPs@Ale-RB-CPC/PEG-Ale nanoparticles generated ^1^O_2_ via efficient Förster resonance energy transfer between the UCNP donor initially in its electronic excited state and the photosensitizer (RB) through nonradiative dipole–dipole coupling [22,23]. Compared to the previously described small spherical UCNPs containing RB [21], the hexagonal particles had a smaller irradiation area and thus a lower DPA absorbance.

### 3.3. Cytotoxicity of Hexagonal UCNP@Ale-RB-CPC/PEG-Ale Particles

**Figure 7:**
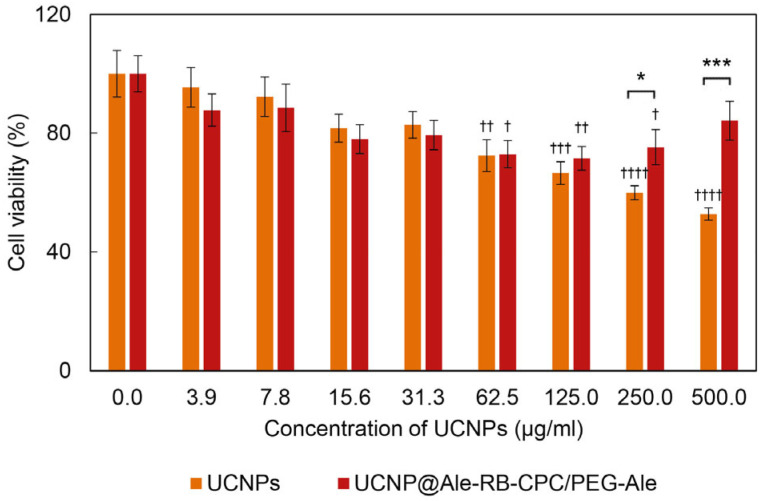
Cytotoxicity of neat hexagonal UCNPs (orange) and UCNP@Ale-RB-CPC/PEG-Ale particles (dark red) incubated with rMSCs for 24 h and measured using the MTT cell viability assay. Error bars represent the standard error of the mean (S.E.M.) calculated from at least three different experiments performed in triplicate; *^,†^ *p* < 0.05, ^††^ *p* < 0.01, ***^,†††^ *p* < 0.001, and ^††††^ *p* < 0.0001; one-way ANOVA with Dunnett’s post hoc test (ꝉ) to compare the particular treatment relative to the control and the two-tailed unpaired Student’s *t*-test (*) to compare differences between the UCNPs and UCNP@Ale-RB-CPC/PEG-Ale.

It is known that UCNPs made from the same core material but of different shapes or sizes can exhibit different cytotoxicity when in contact with living cells [24]. It has also been shown that it is not only the shape or size of the nanoparticles that are responsible for their cytotoxicity but that the first factor that influences the interaction of the particles with the cell plasma membrane is their surface charge [25]; this also determines the possible mechanism of action and/or the severity of cytotoxicity. To evaluate the cytotoxicity of hexagonal UCNP@Ale-RB-CPC/PEG-Ale particles, we selected adult rat stem cells (rMSCs). The mesenchymal stem cells generally tend to be more sensitive to particles or drugs than terminally differentiated cells [26,27]. Cytotoxicity was evaluated by the MTT cell viability assay after 24 h of incubation with UCNPs (Figure 7). The viability of cells in contact with neat hexagonal UCNPs decreased progressively with the increasing particle concentration. From concentrations >125 μg/mL, the particles became toxic according to ISO 10993-5, where cytotoxicity is defined as a reduction in cell viability by more than 30% [28]. The introduction of the Ale-RB-CPC and PEG-Ale coating significantly reduced the cytotoxicity of the particles at the two highest concentrations (250 and 500 µg/mL); the viability increased from 60% to 75% and from 53% to 84%, respectively. As a result, these surface-engineered nanoparticles can be considered non-toxic [28]. Regarding the relationship between cytotoxicity and particle size, hexagonal UCNPs (*D*_h_ = 228 and 720 nm for UCNP and UCNP@Ale-RB-CPC/PEG-Ale, respectively; Table 1) caused higher cytotoxicity than small spherical UCNPs and their RB-modified analogs [21]. These data contradict the literature, where core nanomaterials of the same origin, e.g., gold, silica, polymers, etc., showed lower toxicity for larger particles [29]. However, some studies have also shown that larger nanoparticles induce higher cytotoxicity [30]. The increased cytotoxicity of the neat hexagonal UCNPs was probably due to their high positive surface charge (ξ-potential 38 mV), which is in agreement with previously published results on spherical UCNPs (ξ-potential 36 mV) [21]. Yet, it is known that a positive charge can disrupt the cell membrane [31], enhance nanoparticle uptake [32,33], and also interact with organelles inside cells [34]. Since the RB-CPC and PEG-Ale coating reduced the ξ-potential of the particles to 4 mV, it can be inferred that their positive charge was neutralized, rendering the nanoparticles non-cytotoxic. Experiments with rMSCs have subsequently shown that even the mild toxicity associated with the highest concentration of neat hexagonal UCNPs can be advantageously overcome by a polymer coating and bound RB sensitizer, which on the one hand endowed hexagonal UCNPs with nontoxic properties and on the other hand could enable their prospective use in PDT. Regarding the implications for the *in vivo* applications of UCNPs, it should be noted that upon their addition to biological fluids containing proteins, protein corona formation and particle aggregation, i.e., an increase in their hydrodynamic size, occur. PEG present on the surface of UCNPs@Ale-RB-CPC/PEG-Ale particles reduces the formation of protein coronas, which may affect the size of UCNPs, their charge, aggregation, and intracellular uptake [35,36].

## 4. Conclusions

In this report, the synthesis of a 6-bromohexanoic-acid-modified RB photosensitizer conjugated to hexagonal-shaped UCNP-Ale nanoparticles and the subsequent stabilization by PEG-Ale to yield ^1^O_2_, which can be used in cancer-fighting by photodynamic therapy, is newly described. Compared to the formulation described in our previous work, where small (26 nm) spherical Rose Bengal–ethylphosphonic-acid-conjugated UCNPs were investigated [21], the synthesis conditions were changed to develop relatively large (171 nm) hexagonal particles. In addition, the new treatment of RB with caproic acid was simpler and more convenient compared to the previous method, without the need to dealkylate the intermediate with iodotrimethylsilane. PEG-Ale then represents a promising coating for ensuring the colloidal stability of UCNPs in biomedical applications, since their phosphonate groups have a very strong affinity for the particle surface [14]. Neat UCNPs with a large positive surface charge (38 mV) were cytotoxic at concentrations ≥ 250 µg/mL when interacting with cells. However, neutralization of their charge using PEG-Ale and the bound RB sensitizer allowed the development of non-cytotoxic UCNPs with the future ability to produce singlet oxygen upon irradiation with a 980 nm laser. However, further detailed study of the factors that might influence the cytotoxic properties of UCNPs is necessary for the design and development of safe nanoparticles applicable for PDT.

## Figures and Tables

**Table 1:** Particle size and distribution.

	*D*_n_	*Ð*	*D*_h_ (nm)	*PD*	ξ-Potential (mV)
UCNPs	171	1.01	228	0.04	38
UCNP@Ale			210	0.28	25
UCNP@Ale-RB-CPC			1480	0.15	10
UCNP@Ale-RB-CPC/Ale-PEG			720	0.19	4

*D*_n_—number-average diameter (TEM); *Ð*—dispersity (TEM); *D*_h_—hydrodynamic diameter (DLS); *PD*—polydispersity (DLS).

**Table 2:** XPS analysis of the surface composition of hexagonal UCNP@OA, UCNP@Ale, UCNP@Ale-RB-CPC, and UCNP@Ale-RB-CPC/Ale-PEG particles.

Element.	UCNP@OA	UCNP@Ale	UCNP@Ale-RB-CPC	UCNP@Ale-RB-CPC/Ale-PEG
	(wt.%)			
P 2p	-^a^	5.0	7.4	7.8
Y 3d	38.7	35.9	27.7	21.3
Er 4d	3.2	2.5	1.3	0.7
Yb 4d	0.5	0.5	0.8	0.6
Cl 2p	-	-	1.7	2.3
C 1s C-C, C-H	16.9	14.3	14.2	8.8
C 1s C-O, C-N	-	1.1	5.1	13.4
C 1s C(=O)-NH	-	-	1.1	0.7
C 1s C(=O)-O	2.3	1.2	1.5	1.2
N 1s	-	2.8	2.4	2.0
O 1s	2.9	10.7	16.0	21.0
I 3d	-	-	6.3	4.4
F 1s	27.3	21.3	11.7	11.1
Na 1s	8.2	4.8	2.7	4.7

^a^ Bellow the detection limit of XPS measurement.

## Data Availability

The data presented in this study are available from the first author upon request.

## Supplementary Materials

The following supporting information can be downloaded at: https://www.mdpi.com/article/10.3390/nano13091535/s1. Figure S1: High-resolution ^1^H NMR spectrum of RB-CPC in deuterated methanol at 25 °C.
