# Supplementary material for: Cytotoxicity Evaluation of Photosensitizer-Conjugated Hexagonal Upconverting Nanoparticles
Source: Nanomaterials (Basel). 2023 May 3;13(9):1535. doi: 10.3390/nano13091535 (PMC10180129; doi:10.3390/nano13091535)
Supplement: Supplementary file 1 [file nanomaterials-13-01535-s001.zip › nanomaterials-2377728-supplementary.pdf]

Supplementary Material

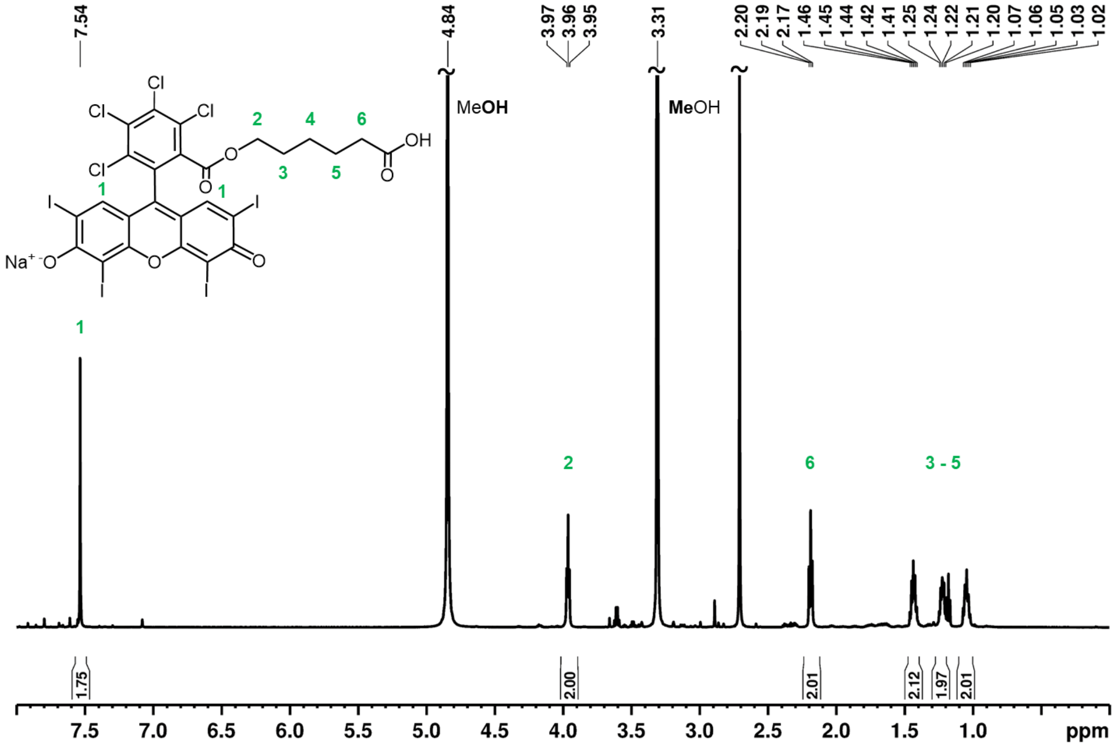

Figure S1. High-resolution ^{1}H NMR spectrum of RB-CPC in deuterated methanol at 25 °C.
